# DETECTING UNDUE HARM ARISING FROM ELDER ABUSE SCREENING: INNOVATIVE METHODS FROM THE EASI-LTC© PILOT STUDY

**DOI:** 10.1093/geroni/igad104.2906

**Published:** 2023-12-21

**Authors:** Stephanie Ballard, Machelle Wilchesky

**Affiliations:** Donald Berman Maimonides Centre for Research in Aging, Montreal, Quebec, Canada; McGill University, Montreal, Quebec, Canada

## Abstract

Screening for elder abuse can cause victims to experience feelings of unpleasantness and/or relive painful memories which can be an ethical concern. Ensuring the safety of all participants/users, in our case long-term care (LTC) residents, is of the utmost importance. Drawing from approaches used in the intimate partner violence and clinical trials literature, we developed a novel typology of harm and a series of procedures to evaluate any negative consequences that might be incurred as a result of participating in the Piloting the Elder Abuse Suspicion Index-long term care©: A Mixed Methods Feasibility Study. Our typology includes subjective and objective reporting from a multitude of sources, including: 1) Interviews with LTC residents using a modified Consequences of Screening Tool, adapted from MacMillan et al, 2009; Opinions of the 2) Study social worker conducting validation assessments and 3) LTC residents and institutional stakeholders; 4) Feedback from study research assistants who administer the tool using a specialized Adverse Events Form; and 5) Chart reviews. Procedures include the creation of an independent interdisciplinary pilot study safety committee, chaired by a geriatric psychiatrist and convened to conduct interim evaluations after the first 5, 10, and 20 residents have participated, the liberation of social work teams, who are to be on standby and available to intervene as necessary, and the creation of participant resource cards displaying pertinent phone numbers for immediate assistance. To our knowledge, this is the first elder abuse study to conduct a thorough analysis of study-induced harm.

